# Role of BMP-4 and Its Signaling Pathways in Cultured Human Melanocytes

**DOI:** 10.1155/2009/750482

**Published:** 2009-12-30

**Authors:** Hee-Young Park, Christina Wu, Mina Yaar, Christina M. Stachur, Marita Kosmadaki, Barbara A. Gilchrest

**Affiliations:** Department of Dermatology, School of Medicine, Boston University, Boston, MA 02118, USA

## Abstract

Bone Morphogenetic Protein (BMP-4) was shown to down-regulate melanogenesis, in part, by decreasing the level of tyrosinase [Yaar et al. (2006) JBC:281]. Results presented here show that BMP-4 down-regulated the protein levels of TRP-1, PKC-*β*, and MCI-R. When paired cultures of human melanocytes were treated with vehicle or BMP-4 (25 ng/ml), MAPK/ERK were phosphorylated within one hour of BMP-4 treatment. Then the activated MAPK/ERK caused an acute phosphorylation of MITF, followed by proteosome-mediated degradation of MITF, the key transcription factor for melanogenic proteins [Wu et al. (2000) Gene & Development:14]. However, prolonged exposure of melanocytes to BMP-4 (up to 48 hours) caused a decrease in the level of MITF-M transcript. In addition, BMP-4 decreased the intracellular level of cAMP, the key regulator of MITF expression. These results demonstrate that BMP-4 activates MAPK/ERK signaling pathway to transiently activate MITF; however, chronic treatment of BMP-4 to melanocytes causes a down-regulation of the expression of MITF, possibly in a cAMP-dependent pathway.

## 1. Introduction

Bone morphogenetic proteins (BMPs) are disulfide-linked dimeric proteins, produced as large precursor proteins (reviewed in [1]). BMPs can be further classified into several subgroups by their amino acid similarities. BMP-2 and BMP-4 have 83% amino acid sequence identity and are the best-studied members in the BMP family (reviewed in [1]). BMP-2 and -4 elicit their biological actions by first binding to their specific cell surface receptors, termed type II receptor (BMPR-II) and type I receptor (BMPR I) [2–6]. Two different types of BMPR-I, termed BMP type IA (BMPR-IA) and type IB (BMPR-IB), have been identified [7–9]. Binding of BMP-2 and BMP-4 to BMPR-II is facilitated by the type I receptors [4, 6]; and upon binding with BMP-2 or -4, BMPR-II subsequently activates type I receptors [10]. Activated BMPR-I phosphorylates Smad-1, -5, or -8 [11], then recruits Smad-4, and the entire complex translocates to the nucleus [12–14] and interacts with specific DNA consensus sequences to either activate or suppress transcription of target genes. Activated BMPR-I can also phosphorylate (activate) extracellular-signal regulated kinase (ERK) via mitogen activated protein kinase (MAPK) and this pathway can also cross-talk with Smads-dependent pathway [15, 16]. 

Recently, it was reported that BMP-4, a member of the TGF-*β* superfamily (reviewed in [17]), negatively modulates melanogenesis in part by reducing the level of tyrosinase expression in cultured human melanocytes [18]. Treatment of quail neural crest cultures with BMP-4 caused a dramatic decrease in melanogenesis [19]. Moreover, in a transgenic mouse model which overexpresses noggin, the known physiological inhibitor of BMP-4 [20], the level of microphthalmia-associated transcription factors (MITF), and binding of *α*-melanocyte stimulating hormone (*α*-MSH) to melanocortin receptor-1 (MC1-R) were increased, resulting in darkening of the coat color [21]. 

The synthesis and dispersion of melanin within the epidermis is largely responsible for skin color as well as for protection from sun-induced injuries. Melanogenesis is initiated when the amino acid tyrosine is oxidized to dopa and dopa quinine by the enzyme tyrosinase, the key and rate-limiting enzyme in melanogenesis (reviewed in [22]). Tyrosinase exists as an inactive form and is activated when protein kinace c-*β* (PKC-*β*) phosphorylates the serine residues in the cytoplasmic domain of this enzyme [23]. Activation of tyrosinase by PKC-*β* was shown to be required for melanogenesis both in vivo [24] and in vitro [23, 25–27], while conversely increased activity and/or expression of PKC-*β* leads to increased pigmentation both in vitro [28] and in vivo [29].

Human melanogenesis is heavily influenced by both paracrine and autocrine factors such as *α*-MSH [30], endothelin-1 [31], interleukin-1 [32], and TNF-*α* [32]. Among these factors, *α*-MSH and endothelin-1 induce pigmentation in part through increased expression and activity of tyrosinase [30, 31, 33]. The expression of both *α*-MSH and endothelin-1 was detected in epidermal keratinocytes and induced when keratinocytes were exposed to ultraviolet irradiation (UV) [31, 34]. Endothelin-1 was shown to induce melanogenesis by activating multiple signaling pathways, including the PKC-dependent pathway [35]. On pigment cells, *α*-MSH first binds to its cell surface receptor MC1-R [36, 37], a heptahelical transmembrane protein coupled to the G-protein [38], and thereby increases the intracellular level of cAMP [33]. One way in which cAMP increases pigmentation is by increasing the expression of MITF [39], shown to be a key transcription factor for melanogenic protein, such as tyrosinase and tyrosinase related protein-1 (TRP-1) and PKC-*β* [40–42]. 

MITF is a basic-helix-loop-helix (bHLH) and leucine zipper transcription factor that binds to conserved consensus elements in gene promoters, specifically the M-(AGTCATGTGCT) and E-(CATGTG) boxes [43], and regulates the transcription of tyrosinase, TRP-1, TRP-2 [41], PKC-*β* [40], and MC1R [44]. MITF can bind as a homodimer or a heterodimer with another related family members (reviewed in [45]). MITF comprises a family of at least nine isoforms [46, 47], of which the MITF-M isoform controls tyrosinase and PKC-*β* transcription in melanocytes [40, 48, 49]. MITF activity and stability are regulated by its phosphorylation state through MAPK/ERK-dependent pathway [50, 51]. Upon phosphorylation, MITF binds to either M-box or E-box consensus sequences to activate transcription [51, 52], but phosphorylation then decreases MITF stability and enhances its degradation in proteosomes [50, 53]. Thus, prolonged activation of MAPK/ERK pathway would result in down-regulation of MITF protein level. More recently, MITF was shown to be a key transcription factor for Rab27a [54], a protein important for melanosome transport, and Pmel17 [48], a protein required for melanosome matrix formation. Therefore, MITF plays a central role in melanin synthesis, as well as melanosome biogenesis and transport.

While factors that increase melanogenesis are well understood, physiological agents that suppress melanogenesis are poorly understood. Recently, it has been shown that interleukin-1*α* and -6, as well as TNF-*α* decrease melanogenesis in human melanocytes [32]. TGF-*β* was also shown to decrease pigmentation [55]. 

In this paper, we further delineate the mechanism by which the BMP-4-dependent signaling pathway down-regulates melanogenesis. Specifically, the intracellular signaling pathways that mediate the effects of BMP-4 and its target genes/proteins are explored. 

## 2. Materials and Methods

### 2.1. Materials

Recombinant human BMP-4 was obtained from R&D Systems, Inc., Minneapolis, MN. Dulbecco's modified essential medium (DMEM), L15, nonessential amino acids, glutamine, Medium 199, and trypsin were purchased from GIBCO/BRL, Gaithersburg, MD. Recombinant basic fibroblast growth factor was purchased from Amgen Inc., Thousand Oaks, CA, bovine pituitary extract (BPE) from Clonetics Corp., San Diego, CA, and hydrocortisone from Calbiochem, San Diego, CA. Insulin, transferrin, and dbcAMP were from Sigma Chemical Co., St. Louis, MO. Nylon membranes and Tag-Ready-to-Go PCR Beads were from Amersham Biosciences, Piscataway, NJ. Bovine calf serum and fetal calf serum were from HyClone Laboratories, Inc., Logan, UT. Polyvinyl difluoride (PVDF) membranes were from BioRad Laboratories, Carlsbad, CA. FuGene6 was purchased from Roche Diagnostic Corp., Nutley, NJ. Lipofectamine Plus reagent was from Invitrogen, Hercules, CA, and promoterless renilla luciferase construct was purchased from Promega, Madison, WI.

### 2.2. Antibodies

Monoclonal antibody specific for PKC-*β* (dilution: 1:50) was purchased from BD Biosciences, San Jose, CA. Polyclonal antibodies against phosphorylated smad 1/5/8 (dilution: 1:10), TRP-1 (dilution: 1:1000), and MCI-R (dilution: 1:100) were purchased from Santa Cruz Biotechnology, Santa Cruz, CA. Monoclonal antibody for tyrosinase (dilution: 1:1000) was purchased from Vector Laboratories, Inc., Burlingame, CA. Monoclonal antibody for MITF (dilution: 1:10) was a generous gift from Dr. David Fisher at the Department of Dermatology, Massachusetts General Hospital, Boston, MA. 

### 2.3. Cells and Media

Neonatal foreskins were used to obtain human melanocytes as previously described [23]. In brief, the epidermis was separated from the dermis after overnight incubation in 0.25% trypsin at 4°C. Primary cultures were then established in Medium 199 containing 1.8 mM Ca^+2^ and supplemented with 10^−9^ M triiodothyronine, 10 *μ*g/mL insulin, 1.4 × 10^−6^ M hydrocortisone, BPE (35 ug/mL), 80 uM dbcAMP, 10 ng/mL basic fibroblast growth factor, and 5–10% fetal bovine serum. All post primary cultures were maintained in low calcium (0.03 mM). Cells at first or second passage were used for all experiments. LH melanoma cells were maintained in DMEM containing 10% of calf serum.

### 2.4. Immunoblot Analysis

Immunoblot analysis was performed as previously described [23]. Cells were harvested in RIPA buffer (0.25 M Tris (pH 7.5), 0.5 M NaCl, 2.5% SDS, and 0.1% Triton-X-100) containing protease inhibitors (100 *μ*M Okadaic acid, 100 *μ*M PMSF, 100 *μ*g/mL Apoprotinin, 2 *μ*g/mL Leupeptin, and 100 *μ*M Sodium Ortho-Vanadate). Protein samples were subjected to 7.5% SDS-PAGE and transferred to a PVDF membrane electrophoretically. The membrane was preincubated in 100% Blotto (5 g nonfat dry milk in 100 mL phosphate buffered saline (PBS)) for 1–3 hours at room temperature with shaking, followed by an overnight incubation with antiserum (0.5–1 ug/mL in 10% Blotto) at 4°C [18, 26, 50]. At the end of the incubation, the membrane was washed extensively with PBS containing 0.5% Tween-20, and processed using the ECL kit. The membrane was then exposed to Kodak X-OMAT film.

### 2.5. Vectors

PKC-*β* promoter-CAT reporter construct and control expression vectors were obtained from Dr. Denise Cooper at the University of South Florida [56].

### 2.6. Transient Transfection

In order to introduce PKC-*β* promoter-CAT reporter construct into LH melanoma cells, 4 ug of DNA were pretreated with Lipofectamine Plus reagent and as an internal control for the transfection efficiency, 0.4 ug of promoterless renilla Luciferase was cotransfected. 24 hours after transfections, cells were treated with vehicle or BMP-4 (25 ng/mL). Cells were then harvested from 48 to 72 hours after BMP-4 treatment. CAT and luciferase assays were performed according to the manufacturer's protocol. 

### 2.7. Semi Quantitative RT-PCR

To determine the effect of BMP-4 on the level of PKC-*β* transcripts, semiquantitative RT-PCR was first performed. Specifically, cDNA was made from total RNA isolated from melanocytes using oligo pd (N)_6_ (Pharmacia Fine Chemicals, Piscataway, NJ). Primers specific for PKC-*β* that recognize both *β*I and *β*II were purchased from Oxford Biomedical Research, Inc. For the internal control, primers against GAPDH (forward: 5′GTCATCATCTCCGCCCCTTC3′ and backward: 3′CCGCACTACCGGCACCCCGT5′) were used. 0.1 ug of cDNA was amplified with 15 pmol of each forward and backward primers. Initially, 37 cycles of amplification were performed on cDNA as well as on RNA from each sample to assure no contamination of RNA samples with genomic DNA. All PCR reactions were amplified for 29 cycles which fell within the exponential phase of the amplification for optimal detection of PKC-*β* and BMP receptors and allowing quantitative analysis of mRNA transcript modulation. Denaturation was performed at 94°C for 30 seconds, primer annealing at 58°C for 1 minute, and DNA polymerization at 72°C for 1 minute in a thermal cycler (MJ Research Inc., Waltham, MA). PCR products were separated over a 1% agarose gel in 1X TAE and stained with ethidium bromide. The levels of PCR products were subjected to the densitometric analysis. PKC-*β* transcript levels were then normalized against the level of GAPDH transcript.

### 2.8. Quantitative Real-Time PCR (qRT-PCR)

The influence of BMP-4 on the level of MITF-M in vehicle vs. BMP-4 treated melanocytes was analyzed using MyiQ^TM^ optical module Real-Time PCR system (Biorad) and iQ SYBR Green Supermix containing the MITF-M upstream primer: 5′CACGGGTCTCTGCTCTCC3′ and downstream primer: 5′GGTTGTTGTTGAAGGTGATGG3′. A second set of MITF-M primers was designed and used to confirm the results: upstream primer: 5′ATAGAAAGTAGAGGGAGGGATAG5′ and downstream primer: 5′TTATTTGCTAAAGTGGTAGAAAGG3′. The PCR reaction was performed in a 50 uL volume containing 2–4 ng cDNA. The final primer and probe concentrations were optimized for each primer/probe combination. Two-step PCR cycling was carried as follows: 95°C 5 minutes × 1 cycle, 95°C 0.5 minute, 55°C 0.5 minute × 40 cycles. As all internal control, GAPDH was amplified in parallel for normalization.

### 2.9. Densitometric Analysis

Densitometric analysis was performed by scanning the autoradiograms of western and northern blots and PCR products into a computer (PC Dell^TM^). Intensity of each scanned band was determined using BIO-RAD Gel Doc 1000/2000 imaging densitometer after background subtraction. 

## 3. Results

### 3.1. BMP-4 Decreased the Protein Levels of TRP-1, PKC-*β*, and MC1-R

**Figure 1:**
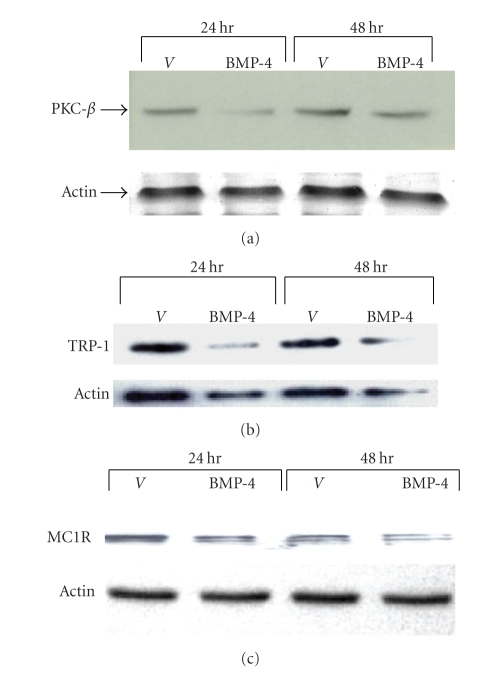
*BMP-4 decreases the protein levels of PKC*-*β*, * TRP-1, and MC1-R*. Paired cultures of melanocytes were treated with either vehicle or BMP-4 (25 ng/mL). Cells were then harvested at 24 and 48 hours for immunoblot analysis using monoclonal antibodies specific for PKC-*β* (a), TRP-1 (b), and MC1-R (c). The protein level of actin was used for the loading control. The level of PKC-*β* protein was decreased by 49.9 ± 29.7% (*P* < .04) at 24 hours and at 48 hours by 47.7 ± 18% (*P* < .04) [23, 27]. The levels of TRP-1 was decreased by 45% ± 5.0% (*P* < .05) and 36 ± 6.5.0% (*P* < .045) at 24 and 48 hours time points, respectively (Figure 1(b)). The protein level of MC1-R was decreased by 50% ± 2.0% (*P* < .03) and 55% ± 2.5% (*P* < .05) at 24 and 48 hours, respectively (Figure 1(c)). A representative result from five separate experiments is shown.

BMP-4 was shown to down-regulate melanogenesis, the key differentiated function of melanocytes, in part by decreasing the expression of tyrosinase [18]. To examine whether BMP-4 affects the expression of other melanogenic proteins, paired cultures of subconfluent primary human melanocytes were treated with vehicle (4 mM HCl/0.01% BSA) or BMP-4 at 25 ng/mL; the optimal concentration to treat melanocytes as previously reported [18], for 24 and 48 hours and the protein levels of TRP-1, PKC-*β*, and MC1-R were assessed, using specific antibodies against these proteins. Results showed that BMP-4 decreased the protein level of PKC-*β* by 49.9 ± 29.7% (*P* < .04) at 24 hours and 47.7 ± 18.0% (*P* < .04) at 48 hours (Figure 1(a)), a magnitude of reduction known to reduce tyrosinase phosphorylation and activity in human melanocytes [23, 27]. The protein level of TRP-1 was decreased by 45% ± 5.0% (*P* < .05) and 36 ± 6.5.0% (*P* < .045) at 24 and 48 hr time points, respectively (Figure 1(b)). The protein level of MC1-R was decreased by 50% ± 2.0% (*P* < .03) and 55% ± 2.5% (*P* < .05) at 24 and 48 hours, respectively (Figure 1(c)). In summary, BMP-4 reduced the protein levels of PKC-*β*, TRP-1, and MC1-R. Earlier time points did not show significant changes in the levels of these proteins in response to BMP-4 treatment.

### 3.2. BMP-4 Activated Both Smads-1/5/8 and MAPK/ERK Signaling Pathways

**Figure 2:**
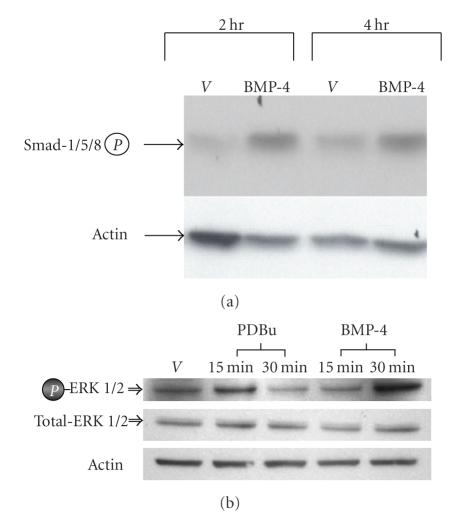
*BMP-4 activates Smad 1/5/8 and MAPK/ERK in cultured melanocytes*. (a) Paired cultures of melanocytes were treated with vehicle or BMP-4 (25 ng/mL) for 2 and 4 hours. At each time point, cells were harvested and immunoblot analysis using polyclonal antibody against phosphorylated Smad 1/5/8 was performed. As the loading control, the membrane was immunoblotted for actin. One representative result from three separate experiments is shown. (b) Paired cultures of melanocytes were treated with vehicle or BMP-4 (25 ng/mL) for 15 and 30 minutes. At each time point, cells were harvested and immunoblot analysis using polyclonal antibody against phosphorylated and nonphosphorylated ERK was performed; PDBu at 1 × 10^−6^ M was used as a control. As the loading control, the membrane was immunoblotted for actin. One representative result from three separate experiments is shown.

It is well documented that BMP-4 regulates transcription of target genes through Smads 1/5/8- [11–14] and/or MAPK/ERK dependent pathways [15, 16]. To examine whether one or both of these pathways are involved in mediating effects of BMP-4 on the expression of melanogenic proteins in human melanocytes, phosphorylation (activation) of Smads 1/5/8 by BMP-4 was first investigated. Paired cultures of melanocytes were treated with vehicle or BMP-4 (25 ng/mL) for 2 and 4 hours as previously done [18]. Then cells were harvested for immunoblot analysis using a polyclonal antibody that reacts against phosphorylated Smads-1/5/8. Results show that Smads-1/5/8 are phosphorylated by BMP-4 within 2 hours of treatment (Figure 2(a)). 

To investigate whether BMP-4 can also activate MAPK/ERK-dependent pathway in human melanocytes, paired cultures of melanocytes were treated with vehicle or BMP-4 (25 ng/mL) for 15 and 30 minutes. Tumor promoter phorbol dibutyrate (PDBu) was used as a positive control as shown before [50]. As shown in Figure 2(b), ERK1/2 was phosphorylated within 30 minutes of BMP-4 treatment. As expected, PDBu phosphorylated ERK1/2 within 15 minutes of treatment. These results suggest that both Smads-1/5/8 and MAPK/ERK-pathways are activated by BMP-4 and may be involved in mediating BMP-4 responses in cultured human melanocytes.

### 3.3. Level of Phosphorylated MITF is Transiently Increased by BMP-4

**Figure 3:**
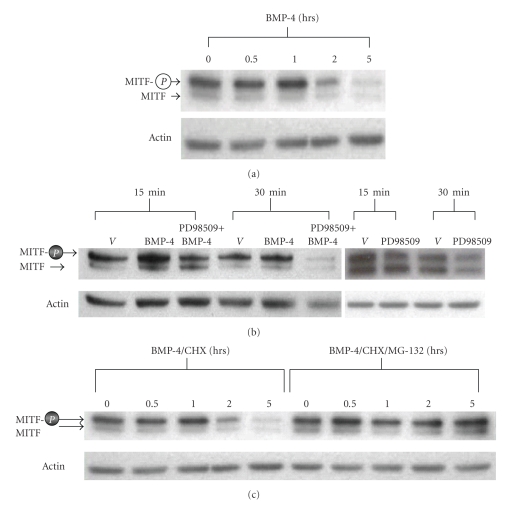
*Effects of BMP-4 on the level of phosphorylated level of MITF*. (a) Paired cultures of melanocytes were treated with BMP-4 (25 ng/mL) for 0, 0.5, 1, 2, and 5 hours. At each time point, cells were harvested and immunoblot analysis using monoclonal antibody against MITF was performed (provided by Dr. David Fisher from the Dana Farber Cancer Institute). This antibody has been shown to recognize both phosphorylated and nonphosphorylated MITF. As the loading control, the membrane was immunoblotted for actin. One representative result from three separate experiments is shown. (b) Paired cultures of melanocytes were treated with BMP-4 (25 ng/mL) for 15 and 30 minutes in presence or absence of MEK inhibitor PD98509 at 10 *μ*m. At each time point, cells were harvested and the level of MITF protein was determined. One representative result from three separate experiments is shown. (c) Paired cultures of melanocytes were treated with BMP-4 (25 ng/mL) and cycloheximide (15 ug/mL) for 0, 0.5, 1, 2, and 5 hours in presence or absence of proteosome inhibitor MG-132 at 10 *μ*m. At each time point, cells were harvested and the level of MITF protein was determined using immunoblot analysis. One representative result from three separate experiments is shown.

It is well known that activated MAPK/ERK pathway can phosphorylate MITF, which results in a transient activation and then degradation of MITF [50, 51]. MITF is the key transcription factor that regulates the expression of TRP-1, PKC-*β*, and MC1-R. Therefore, it is possible that the effect of BMP-4 on the expression of these melanogenic proteins is mediated in part or totally through MITF. To test whether BMP-4 treatment causes phosphorylation of MITF, paired cultures of melanocytes were treated with BMP-4 (25 ng/mL) for 0, 0.5, 1, 2, and 5 hours. Results show a high basal level of phosphorylated MITF (0 hr). Then the level of phosphorylated MITF increased by a 2–4 fold (*P* < .03) at 1 hour, and there was a subsequent reduction at 5 hours time point (Figure 3(a)). To confirm that phosphorylation of MITF by BMP-4 treatment is mediated through MAPK/ERK-dependent pathway, paired cultures of melanocytes were treated with MAKP inhibitor PD98056 (10 uM) or vehicle (DMSO). PD98056 completely blocked the BMP-4 induced MITF phosphorylation (Figure 3(b)), confirming that BMP-4 phosphorylates MITF through MAPK/ERK-dependent pathway. 

Since it is well known that phosphorylation of MITF by MAPK/ERK-dependent pathway results in the proteosome-mediated degradation of MITF [50, 51], the decreased level of MITF by BMP-4 treatment at 4 to 5 hours is likely due to degradation of MITF. To further confirm this possibility, paired cultures of melanocytes were treated with BMP-4 (25 ng/mL) for 0, 1, 2, 3, and 5 hours in presence or absence of proteosome inhibitor MG-132 (10 uM). Protein synthesis inhibitor cyclohexamide (15 ug/mL) was added to all treatment groups. Results show that BMP-4 first increased the level of phosphorylated MITF (0.5 to 1 hour) as expected and then the level of MITF began to decline at 2 hours and was significantly reduced at 5 hours. Presence of the proteosome inhibitor MG-132 completely blocked the degradation of MITF (Figure 3(c)). These results clearly demonstrate that BMP-4 transiently phosphorylated MITF, thus potentially promote acute melanogenesis, followed by down-regulation.

### 3.4. Chronic Treatment of BMP-4 Decreased Total MITF

**Figure 4:**
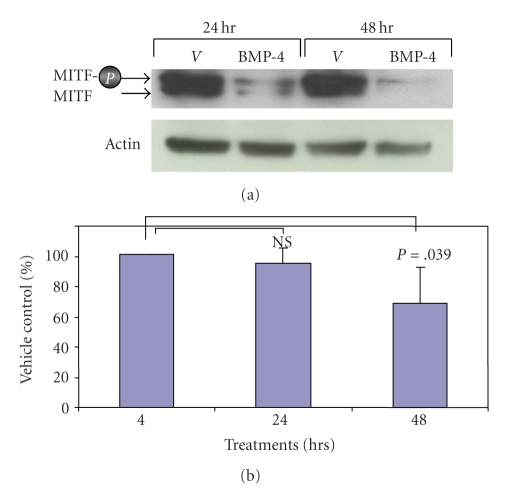
*BMP-4 reduces the level of total MITF protein*. (a) Paired cultures of melanocytes were treated with vehicle or BMP-4 for 24 and 48 hours. At each time point, cells were harvested and immunoblot analysis using monoclonal antibody against MITF was performed. As the loading control, the membrane was immunoblotted for actin. One representative result from three separate experiments is shown. (b) In parallel, total RNA was isolated and quantitative Real-Time PCR was performed using specific primers against MITF-M as described under the Materials and Methods. Paired student t-test was performed for the statistical analysis.

If BMP-4 causes a transient phosphorylation of MITF, followed by proteosome-mediated degradation, then a long term treatment of BMP would ultimately result in a decreased level of total MITF. To test this possibility, paired cultures of melanocytes were treated with BMP-4 (25 ng/mL) or vehicle alone for 24 or 48 hours. Immunoblot analysis using a specific monoclonal antibody against MITF that detects both phosphorylated and nonphosphorylated MITF revealed that BMP-4 dramatically decreased the levels of both forms of MITF at both 24 and 48 hours time points (Figure 4(a)). 

To further examine whether the decreased level of MITF by chronic treatment of melanocytes with BMP-4 is entirely due to the proteosome-mediated degradation of MITF or BMP-4 could also reduce the level of MITF transcripts, paired cultures of melanocytes were treated with vehicle or BMP-4 (25 ng/mL) for 4, 24, and 48 hours and total RNA was isolated. RNA was reverse transcribed into cDNA and the level of MITF-M transcripts between vehicle treated and BMP-4 treated samples was determined using quantitative Real-Time PCR. The level of GAPDH transcript was used as the internal control. The MITF-M transcript level remained unchanged at 4 and 24 hours after the BMP-4 treatment (Figure 4(b)). However, after 48 hours of BMP-4 treatment, the level of MITF-M transcript was significantly (*P* = .039) decreased (Figure 4(b)). These results suggest that the decreased protein level of MITF by BMP-4 is initially due to the proteosome-mediated degradation. BMP-4 then subsequently decreased the expression of MITF at the transcriptional level.

### 3.5. BMP-4 Reduces the Intracellular Level of cAMP in Melanocytes

**Figure 5:**
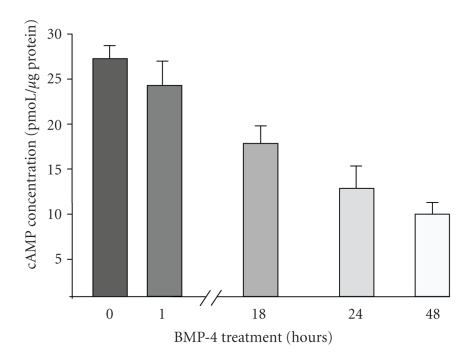
*BMP-4 decreases the intracellular level of cAMP*. Paired cultures of melanocytes were treated with BMP-4 for 0, 1, 18, 24, and 48 hours. At each time point, cells were harvested and the level of intracullelar cAMP level was determined. One representative result from three separate experiments is shown.

Results thus far suggest that BMP-4 transiently activates MITF through MAPK/ERK-dependent pathway. Our results also suggest that BMP-4 transcriptionally regulates the expression of MITF. Recent reports have suggested that cAMP-dependent pathway may mediate BMP-4 induced changes [57]. Because cAMP has been implicated to be the key molecule inducing pigmentation [39, 41], in part through inducing the expression of MITF-M [40], possible effects of BMP-4 on the level of intracellular cAMP in cultured human melanocytes were explored. Paired cultures of subconfluent melanocytes were treated with BMP-4 (25 ng/mL). Then cells were harvested at each time point and the level of cAMP was measured using enzyme-linked immunoblot assay (ELISA). Within 18 hours of BMP-4 treatment, cAMP level began to decrease and by 48 hours, BMP-4 significantly reduced the intracellular level of cAMP (Figure 5). This result suggests that part of BMP-4 effects on the expression of MITF may be mediated through cAMP-dependent pathway.

### 3.6. Effect of BMP-4 on the Promoter Activity of PKC-*β*

It is not clear to what extent Smads-1/5/8 pathway is involved in mediating the effects of BMP-4 on the expression of TRP-1, PKC-*β*, MC1-R, and MITF. Published nucleotide sequences of the promoter regions of tyrosinase, and PKC-*β* revealed that they both contain consensus sequences for Smad-1 and Smad-5 [58, 59], suggesting that BMP-4 could regulate the expression of these two key melanogenic proteins through Smad-dependent pathway. 

**Figure 6:**
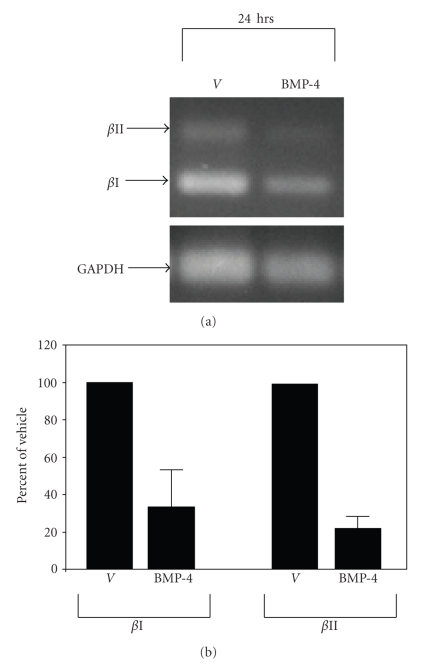
*BMP-4 decreases the levels of PKC*-*β in mRNA in LH melanoma cells*. Quantitative Real-Time PCR was performed using the same cDNA used in semi-quantitative RT-PCR (a). The level of PKC-*β* transcripts were normalized using the level of GAPDH transcripts. Results were then expressed as the percent of the vehicle-treated samples (b). In three independent experiments, BMP-4 significantly decreased the level of PKC-*β* transcripts (*P* < .03).

To explore the possibility that BMP-4 may regulate PKC-*β* expression, in part, by suppressing the promoter activity of the PKC-*β* gene, presumably through Smad-dependent pathway, the level of PKC-*β* transcripts was first examined using semiquantitative RT PCR. As shown in Figure 6(a), two transcripts of PKC-*β* were identified and the levels of both transcripts were significantly decreased by BMP-4 (Figure 6). 

**Figure 7:**
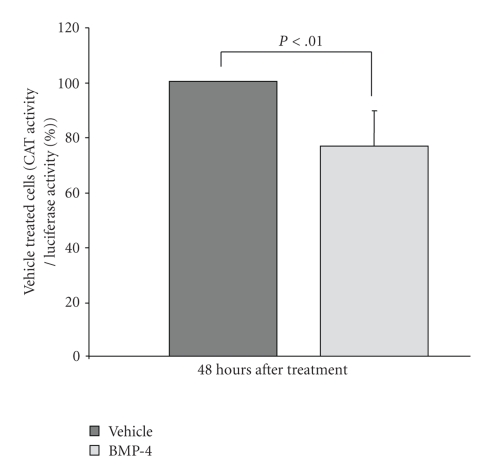
*Effects of BMP-4 on the promoter activity of PKC*-*β*. In order to determine whether BMP-4 affects promoter activity of PKC-*β*, paired cultures of LH melanoma cells were transfected with PKC-*β* promoter CAT reporter construct along with promoter-less renilla luciferase to assess the transfection efficiency, and treated with BMP-4 for 48 hours. Cells were harvested and CAT and Luciferase activities were performed. A total of eight separate transfections were performed, data were pulled and expressed as a percent of vehicle-treated samples. BMP-4 decreased the promoter activity of PKC-*β* by 25 ± 5% (*P* < .01).

To demonstrate whether BMP-4 affects the promoter activity of PKC-*β*, PKC-*β* promoter-CAT reporter construct [56] was transfected into LH human melanoma cells that were originally cultured from a metastatic melanoma [60]. Unlike many other melanoma lines, in which the endogenous expression of PKC-*β* is lost [61, 62], LH human melanoma cells retain the expression of PKC-*β*, and express both BMPR-IA/IB and BMPR-II (data not shown). Moreover, the expression of PKC-*β* in LH melanoma cells respond to BMP-4 similar to those found in cultured melanocytes where both the protein and the mRNA of PKC-*β* were reduced by treating the cells with BMP-4 (25 ng/mL) for 24 hours (data not shown). Then, to examine whether BMP-4 suppresses the promoter activity of PKC-*β*, a PKC-*β* promoter-CAT reporter construct was transfected into paired cultures of LH melanoma cells. A promoterless renilla-luciferase reporter construct was cotransfected into each plate to normalize the transfection efficiency among plates, and results were expressed as CAT activity per luciferase activity. Plates were treated with BMP-4 (25 ng/mL) or vehicle alone 24 hours after transfection, and cells were harvested 48 hours after BMP-4 treatment. In eight separate experiments, the promoter activity of PKC-*β* was reduced by ~25 ± 5% in BMP-4-treated cells when compared to vehicle-treated cells (Figure 7). A paired Student t-test showed that suppression of the promoter activity of PKC-*β* by BMP-4 was statistically significant (*P* < .01). Although the effect of BMP-4 on the promoter activity of PKC-*β* was statistically significant, the total level of reduction was modest and less than the reduction level seen in both PKC-*β* mRNA and protein levels. These results suggest that BMP-4 utilizes only partially Smads-dependent pathway to regulate the expression of PKC-*β*.

## 4. Discussion

Bone morphogenetic proteins (BMPs) were shown to be important for the development of melanoblasts during embryogenesis (reviewed in [19]). In recent years, it has been shown that BMPs, BMP 2/4 in particular, may play key roles in the biological function of differentiated epidermal melanocytes as a paracrine and/or autocrine factor by influencing the activities of melanocytes. Both epidermal keratinocytes and melanocytes express BMP-4 and its receptors; BMPR-IA, BMPR-IB, and BMPR-II, and both BMP-4 and all three BMP-4 receptors are down-regulated in these cells when exposed to UV [18]. Moreover, BMP-4 and its signaling pathway have recently been suggested to be a novel modulator of human pigmentation [18]. 

BMP-4 is able to exert its biological action through Smads- and/or MAPK/ERK signaling pathways [15, 16]. Our results showed that BMP-4 treatment caused activation of both signaling pathways. However, results strongly suggest that BMP-4 preferentially utilizes MAPK/ERK-pathway to regulate synthesis of melanin or melanogenesis once it binds to its cell surface receptors. Experiments presented in this paper demonstrate that one of the major target proteins of BMP-4 activated MAPK/ERK pathway in human melanocytes is MITF. Modulation of activity and/or expression of MITF could have a significant impact on the biology of melanocytes because MITF is the central transcription factor for many of the melanogenic proteins, including tyrosinase [41], TRP-1, [41] PKC-*β* [40–42], and MC1R [44], as well as antiapoptotic protein bcl2 [63]. When BMP-4 is added to the melanocyte cultures, MITF is transiently phosphorylated by BMP-4 via MAPK/ERK-dependent signaling pathway and this phosphorylation initially activates the transcriptional activity of MITF, as previously shown [50, 51]. Therefore, BMP-4 can acutely promote melanogenesis. However, the phosphorylated MITF results in proteosome-mediated degradation, as previously reported [53]. The eventual decreased level of MITF by BMP-4 would then cause an overall decrease in the levels of melanogenic proteins and thus, cause down-regulation of melanogenesis. 

Cell surface receptor c-kit, known to be activated after the interaction with its ligand Stem cell factor (CSF), was one of the first cell surface receptors on melanocytes that was shown to activate MAPK/ERK pathway that causes the transient phosphorylation of MITF and thus, activation of this transcription factor [51]. It was recently shown that c-kit has a soluble form that is released from melanocytes and sequesters CSF and therefore, inhibits melanogenesis [64]. It would be interesting to determined whether BMP-4 and soluble c-kit could work synergistically to down-regulate the level of MITF. 

Our results further suggest that effects of BMP-4 on the expression of TRP-1, PKC-*β*, and MC1-R are mainly mediated indirectly through decreasing the level of MITF. Decreased level of MITF would then lead to a decreased level of transcriptional activities of these genes. In fact, it caused a reduction in the level of the transcripts of PKC-*β*. However, part of reduction in PKC-*β* transcripts by BMP-4 is through Smad-dependent pathway, as explained later. Nonetheless, MAPK/ERK-dependant pathway appears to play a larger contribution to the reduced level of PKC-*β* through MITF. 

Our results further suggest that BMP-4 down-regulates the protein level of MITF first by proteosome-mediated degradation. It is then followed by a decrease in the level of MITF-M transcripts, suggesting that BMP-4 can transcriptionally down-regulate the expression of MITF-M. This decrease in the level of MITF-M may be mediated by cAMP-dependent pathway. Results showed that BMP-4 decreased the intracellular level of cAMP. It has been reported that BMP-4 can regulate the intracellular level of cAMP [19, 65, 66]. In melanocytes it is not clear whether BMP-4 down-regulates the intracellular level of cAMP through down-regulating the expression of MC1-R, or by other mechanism. It is unlikely that Smads-dependent pathway is involved in down-regulation of MITF-M transcripts because the promoter region of MITF gene does not contain DNA consensus sequences for Smads binding sites [46]. 

MITF was also shown to positively regulate the expression of the antiapoptotic protein bcl2 [63]. Therefore, decreased level of MITF by BMP-4 could eventually affect the survival of melanocytes. Yaar et al. have reported that short-term exposure of BMP-4 to melanocytes did not change the proliferation rate and it did not cause apoptosis of melanocytes [18]. However, BMP-4 could potentially increase the susceptibility of melanocytes to damages such as UV and other chemical agents that could reduce survival of melanocytes. Therefore, role of BMP-4 on melanocyte may have implications beyond its role in melanogenesis. 

Smads 1, 5, and/or 8 were phosphorylated by BMP-4 within 2 hours of treatment in cultured human melanocytes. Moreover, Yaar et al. have shown that activated (phosphorylated) Smad 1, 5, and/or 8 translocates to nucleus [18]. Among the melanogenic proteins examined, only the promoter regions of PKC-*β* reveal twelve Smad-1 and one Smad-5 consensus sequences [58], suggesting that BMP-4 could directly regulate the transcriptional activity of the PKC-*β* gene. Transfection of a PKC-*β* promoter–CAT reporter construct into LH cells showed modest but significant suppressions of PKC-*β* promoter–CAT reporter activity. The level of decrease in PKC-*β* transcript by BMP-4 was greater than the suppression of the PKC-*β* promoter activity. These results suggest that the effect of BMP-4 on PKC-*β* expression may involve direct transcriptional regulation. However, clear demonstration of direct interaction between PKC-*β* promoter and Smads 1, 5, and/or 8 would require additional experiments involving gel-shifts and/or CHIP assays. 

In murine melanoma cells, TGF-*β* decreases pigmentation by decreasing the stability of tyrosinase mRNA and protein [67], as well as decreasing the level and the activity of MITF [55]. TGF-*β* was shown to decrease only the level of phosphrylated MITF [55]. However, results presented in this paper show that the levels of both phosphorylated and nonphosphorylated MITF were decreased by BMP-4 in cultured human melanocytes. Even though BMP-4 belongs to the superfamily of TGF-*β*, their detailed mechanisms of action may differ with regard to melanogenesis.

The findings of the present in vitro studies are likely of great in vivo relevance, as transgenic mice in which BMP-4 action is antagonized by overexpression of its physiologic inhibitor noggin in hair follicles have a markedly darker coat color [21]. 

Our results suggest that the BMP-4-dependent pathway is a key physiologic negative modulator of melanogenesis, suppressing the expression of melanogenic proteins and signaling molecules. BMP-4 may thus provide a novel target for modulation of melanogenesis.

## Abbreviations

BMP-4: Bone-morphogenetic protein 4
PKC-*β*: Protein kinase C-*β*
MITF: Microphthalmia-associated transcription factor
CAT: Chloramphenicol acetyl transferase
DMEM: Dulbecco's modified essential medium
BPE: Bovine pituitary extract
TRP-1: Tyrosinase related protein-1
MC1-R: Melanocortin receptor 1
MAPK: Mitogen-activated protein kinase
ERK: Extracellular-signal regulated kinase
UV: Ultraviolet irradiation.
